# Antibacterial Co(II), Cu(II), Ni(II) and Zn(II) Complexes of Thiadiazoles Schiff Bases

**DOI:** 10.1155/MBD.2001.95

**Published:** 2001

**Authors:** Zahid H. Chohan, Maimoon F. Jaffery, Claudiu T. Supuran

**Affiliations:** 1 Department of Chemistry Islamia University Bahawalpur Pakistan; 2 Università degli Studi Laboratorio di Chimica Bioinorganica Via Gino Capponi 7 Firenze 50121 Italy

## Abstract

Schiff bases were obtained by condensation of 2-amino-l,3,4-thiadiazole with 5-substituted-salicylaldehydes which were further used to obtain complexes of the type [M(L)_2_]Cl_2_, where M=Co(II), Cu(II), Ni(II) or Zn(II). The new compounds described here have been characterized by physical, spectral and analytical data, and have been screened for antibacterial activity against several bacterial strains such as *Escherichia coli*, *Staphylococcus aureus*, and *Pseudomonas aeruginosa*. The antibacterial potency of these Schiff bases increased upon chelation/complexation, against the tested bacterial species, opening new aproaches in the fight against antibiotic resistant strains.


### ANTIBACTERIAL Co(II), Cu(II), Ni(II) AND Zn(II) COMPLEXES OF THIADIAZOLES SCHIFF BASES

Zahid H. Chohan*[1], Maimoon F. Jaffery[1] and Claudiu T. Supuran[2]

[1]Department of Chemistry, Islamia University, Bahawalpur, Pakistan

[2]Università degli Studi, Laboratorio di Chimica Bioinorganica,

Via Gino Capponi 7, 50121 Firenze, Italy

### ABSTRACT
Schiff bases were obtained by condensation of 2-amino-1,3,4-thiadiazole with 5-substituted-salicylaldehydes which were further used to obtain complexes of the type $[M(L)_2]Cl_2$, where M=Co(II), Cu(II), Ni(II) or Zn(II). The new compounds described here have been characterized by physical, spectral and analytical data, and have been screened for antibacterial activity against several bacterial strains such as *Escherichia coli*, *Staphylococcus aureus*, and *Pseudomonas aeruginosa*. The antibacterial potency of these Schiff bases increased upon chelation/complexation, against the tested bacterial species, opening new aproaches in the fight against antibiotic resistant strains.

### INTRODUCTION
Many studies stressed the role of metal ions in important biological processes, whereas the inorganic pharmacology started to be an important field *per se*, with more than 25 inorganic compounds, being used in therapy as antibacterial, antiviral and anticancer drugs[1-7]. Kirschner *et al* have suggested[8] that the transfer of the metal ion from the ligand to the cancer-associated viruses was an important mechanism for designing new anticancer therapies. The inverse process, i.e., coordinating a metal ion from an important biomolecule, such as for instance a zinc finger protein, has recently been used to design novel antiviral therapies, targeted against human immunodeficiency (HIV) and human papilloma virus (HPV) infections.[9] Palladium and platinum complexes of 6-mercaptopurine have already been shown to destroy[10] the adenocarcinomas, whereas the complexes of dialkyldithiophosphate[11], cisplatin and carboplatin are potent antineoplastic drugs used in chemotherapy[12]. They show their best results in the treatment of testicular and ovarian carcinoma and are also effective against bladder tumors and tumors of head and neck. [12] It has also been demonstrated that chelation/complexation tend to make biologically inactive compounds active[13-16]. All these evidences however, need to highlight more the biological application of chelation in therapeutic potentials. For this purpose, keeping in view the antibacterial[17,18], antiviral[19], antifungal[20,21], antitumor[22-24] and antileukemia[25] properties of some thiadiazole derivatives, we report here some new Schiff bases, obtained by the condensation reaction of 2-amino-1,3,4-thiadiazole with 5-substituted salicyloaldehydes ($HL^1$-$HL^3$) (Fig. 1). These newly prepared Schiff bases were used to synthesize their Co(II), Cu(II), Ni(II) and Zn(II) complexes. In order to evaluate and establish the role of metal ions on the antibacterial activity, these Schiff bases in comparison to their metal(II) complexes were screened for antibacterial activity against bacterial species such as *Escherichia coli*, *Staphylococcus aureus*, and *Pseudomonas aeruginosa*, all of which ultimately developed increasing resistance to the classical antibiotics.[26]

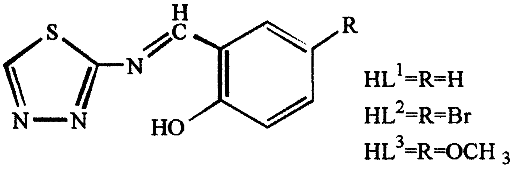

Fig.1. Structure of the Schiff bases

### EXPERIMENTAL
### Material and Methods
All chemicals and solvents used were of Analar grade. All metal salts were used as chlorides. IR spectra were recorded on a Philips Analytical PU 9800 FTIR spectrophotometer as KBr discs. UV-Visible spectra were obtained in DMF on a Hitachi U-2000 double-beam spectrophotometer. C, H and N analyses was carried out by Butterworth Laboratories Ltd. Conductance of the metal complexes was determined in DMF on a Hitachi YSI-

32 model conductometer. Magnetic measurements were made on solid complexes using the Gouy method. Melting points were recorded on a Gallenkamp apparatus and are uncorrected.

**Preparation of Schiff base ($HL^1$)**

Salicylaldehyde (1.2 g, 1.1 mL, 0.01 M) in ethanol (10 mL) was added to a hot ethanol solution (20 mL) of 2-amino-1,3,4-thiadiazole (1.0 g, 0.01 M). Then 2-3 drops of conc.$H_2SO_4$ were added and mixture refluxed for 2 h. On cooling, a yellow solid product was formed which was filtered, washed with ethanol, then with ether and dried. Crystallization in hot ethanol gave $HL^1$ (1.0g, 80 %), M.p 134°C. The same method was applied for the preparation of $HL^2$ (1.5 g, 70 %), M.p 115°C and $HL^3$ (1.6 g, 65 %), M.p 128°C by using their respective reagents in the same molar ratio.

**Preparation of cobalt(II) Complex of $HL^1$**

A warm ethanol solution (20 mL) of $HL^1$ (0.6 g, 0.002 M) was added to a magnetically stirred solution of cobalt chloride hexahydrate (0.24 g, 0.001 M) in distilled water (25 mL). The mixture was refluxed for 1 h and cooled to room temperature. On cooling, pink precipitates were formed which were filtered, washed with ethanol, acetone and ether, and dried by suction. Crystallization in aqueous ethanol (70 : 30) gave the desired metal complex (1) (0.5 g, 68 %). All other metal complexes were formed respectively following the same method.

**Antibacterial Studies**

The synthesized metal complexes, in comparison to the uncomplexed Schiff bases were screened for their antibacterial activity against pathogenic bacterial species, *Escherichia coli, Staphylococcus aureus* and *Pseudomonas aeruginosa*. The paper disc diffusion method[26-28] was adopted for the determination of antibacterial activity.

### RESULTS AND DISCUSSION

**Physical Properties**

The Schiff bases ($HL^1$-$HL^3$) (Fig. 1) were prepared by refluxing an appropriate amount of 2-amino-1,3,4-thiadiazole with the respective salicylaldehydes in ethanol in 1 : 1 molar ratio. The structures of these Schiff bases were established with the help of their IR, NMR, and microanalytical data (Tables 1 and 2). All metal complexes [(1)-(16)] (Table 3) of these Schiff bases were air stable and prepared by the stoichiometric reaction of the respective metals chlorides with the Schiff base in molar ratio M:L of 1:2. The complexes are intensely colored and amorphous solids, which decompose above 200° C. They are insoluble in common organic solvents such as ethanol, methanol, chloroform or acetone being only soluble in DMSO and DMF. Molar conductance values of the soluble complexes in DMF show low values (14-22 $ohm^{-1}cm^2mol^{-1}$) indicating[29] that they are all non-electrolytic in nature.

Table I. Spectral and Analytical Data of the Schiff bases

| Schiff base | IR ($cm^{-1}$) | $^1H$ NMR (DMSO-$d_6$) ppm | $^{13}C$ NMR (DMSO-$d_6$) (ppm) | Calc (Found) % C H N |
|---|---|---|---|---|
| $HL^1$ $C_9H_7N_3OS$ [205.1] | 3425 (br, OH), 1630 (s, HC=N), 1620 (s, C=N). | 8.7 (s, 1H, heteroaromatic), 7.4 (s, 1H, CH=N), 6.8 (m, 1H, Ph), 7.2 (m, 1H, Ph), 7.4 (m, 1H, Ph), 7.7 (m, 1H, Ph), 9.9 (s, 1H, OH). | 116.4, 118.2, 118.5 (aromatic), 131.1, 132.2, 160.5 (aromatic), 143.1, 156.1 (heteroaromatic), 165.2 (HC=N). | 52.7 3.4 20.5 (52.9) (3.2) (20.8) |
| $HL^2$ $C_9H_6BrN_3OS$ [284.0] | 3430 (br, OH), 1635(s, HC=N), 1620 (s, C=N). | 8.7 (s, 1H, heteroaromatic), 7.5 (s,1H,CH=N), 7.3 (m, 1H, Ph), 7.6 (m, 1H, Ph), 7.8 (m, 1H, Ph), 10.1 (s, 1H, OH). | 116.5, 118.1, 118.6 (aromatic), 131.5, 132.3, 160.5 (aromatic), 143.3, 155.8 (heteroaromatic), 165.2 (HC=N). | 38.0 2.1 14.8 (38.4) (2.0) (14.5) |
| $HL^3$ $C_{10}H_9N_3O_2S$ [235.1] | 3432 (br, OH), 1635 (s, HC=N), 1625 (s, C=N). | 3.6 (s, 3H, $OCH_3$, 8.8 (s, 1H, heteroaromatic), 7.6 (s, 1H, CH=N), 7.4 (m, 1H, Ph), 7.6 (m, 1H, Ph), 7.8 (m, 1H, Ph), 10.2 (s, 1H, OH). | 31.7 ($OCH_3$), 116.5, 118.2, 118.6 (aromatic), 131.7, 132.1, 160.5 (aromatic), 143.2, 156.3 (heteroaromatic), 165.1 (HC=N). | 51.0 3.8 17.9 (51.3) (3.9) (17.6) |

Table 2. Physical and Analytical Data of the Metal(II) Chelates

| No | Metal chelate/<br>Mol. Formula | Yield<br>(%) | M.p ($^{o}C$)<br>(decomp) | B.M.<br>($\mu_{eff}$) | Calc (Found)%<br>C H N |
|---|---|---|---|---|---|
| 1 | $[Co(L^1)_2]Cl_2$ [537.9]<br>$C_{18}H_{12}CoCl_2N_6O_2S_2$ | 60 | 222-224 | 4.2 | 40.2 2.2 15.6<br>(40.6) (2.1) (15.4) |
| 2 | $[Co(L^2)_2]Cl_2$ [695.7]<br>$C_{18}H_{10}CoCl_2Br_2N_6O_2S_2$ | 56 | 220-222 | 4.7 | 31.0 .1.4 12.1<br>(31.3) (1.4) (12.3) |
| 3 | $[Co(L^3)_2]Cl_2$ [597.9]<br>$C_{20}H_{16}CoCl_2N_6O_4S_2$ | 58 | 221-223 | 4.5 | 40.1 2.7 14.0<br>(40.5) (2.8) (14.2) |
| 4 | $[Cu(L^1)_2]Cl_2$ [542.5]<br>$C_{18}H_{12}CuCl_2N_6O_2S_2$ | 61 | 225-227 | 1.3 | 39.9 2.2 15.5<br>(40.2) (2.3) (15.2) |
| 5 | $[Cu(L^2)_2]Cl_2$ [700.3]<br>$C_{18}H_{10}CuCl_2Br_2N_6O_2S_2$ | 57 | 218-220 | 1.6 | 30.8 1.4 12.0<br>(30.5) (1.0) (12.3) |
| 6 | $[Cu(L^3)_2]Cl_2$ [602.5]<br>$C_{20}H_{16}CuCl_2N_6O_4S_2$ | 58 | 218-220 | 1.4 | 39.8 2.7 13.9<br>(39.6) (2.9) (13.4) |
| 7 | $[Ni(L^1)_2]Cl_2$ [537.7]<br>$C_{18}H_{12}NiCl_2N_6O_2S_2$ | 58 | 212-214 | 3.1 | 40.2 2.2 15.6<br>(40.6) (2.5) (15.4) |
| 8 | $[Ni(L^2)_2]Cl_2$ [695.5]<br>$C_{18}H_{10}NiCl_2Br_2N_6O_2S_2$ | 60 | 226-228 | 3.4 | 31.1 1.4 12.1<br>(31.0) (1.6) (12.3) |
| 9 | $[Ni(L^3)_2]Cl_2$ [597.7]<br>$C_{20}H_{16}NiCl_2N_6O_4S_2$ | 61 | 220-222 | 3.3 | 40.2 2.7 14.1<br>(40.5) (2.4) (14.0) |
| 10 | $[Zn(L^1)_2]Cl_2$ [544.4]<br>$C_{18}H_{12}ZnCl_2N_6O_2S_2$ | 56 | 215-218 | Dia | 39.7 1.8 15.4<br>(39.2) (1.7) (15.9) |
| 11 | $[Zn(L^2)_2]Cl_2$ [702.2]<br>$C_{18}H_{10}ZnCl_2Br_2N_6O_2S_2$ | 58 | 222-224 | Dia | 30.1 1.4 12.0<br>(30.5) (1.9) (12.1) |
| 12 | $[Zn(L^3)_2]Cl_2$ [604.4]<br>$C_{20}H_{16}ZnCl_2N_6O_4S_2$ | 60 | 218-220 | Dia | 39.7 2.6 13.9<br>(39.5) (2.3) (13.6) |

Table 3. IR and UV-Visible Spectral Data of the Metal(II) Chelates

| No | IR ($cm^{-1}$) | $\lambda_{max}$ ($cm^{-1}$) |
|---|---|---|
| 1 | 1625 (HC = N), 1600 (C = N),<br>525 (M-N), 455 (M-O) | 30210, 18425, 8815 |
| 2 | 1620 (HC = N), 1595 (C = N),<br>525 (M-N), 455 (M-O) | 30575, 17850, 8665 |
| 3 | 1620 (HC = N), 1600 (C = N),<br>530 (M-N), 460 (M-O) | 30545, 17950, 8810 |
| 4 | 1620 (HC = N), 1600 (C = N),<br>530 (M-N), 460 (M-O) | 30550, 22152 |
| 5 | 1625 (HC = N), 1600 (C = N),<br>525 (M-N), 460 (M-O) | 30645, 22355 |
| 6 | 1620 (HC = N), 1595 (C = N),<br>530 (M-N), 455 (M-O) | 30575, 22315 |
| 7 | 1625 (HC = N), 1600 (C = N),<br>525 (M-N), 460 (M-O) | 28540, 15945, 10145 |
| 8 | 1625 (HC = N), 1600 (C = N),<br>530 (M-N), 455 (M-O) | 29210, 16250, 9815 |
| 9 | 1620 (HC = N), 1595 (C = N),<br>525 (M-N), 455 (M-O) | 28880, 16175, 9910 |
| 10 | 1620 (HC = N), 1595 (C = N),<br>525 (M-N), 460 (M-O) | 28450 |
| 11 | 1625 (HC = N), 1600 (C = N),<br>525 (M-N), 460 (M-O) | 28475 |
| 12 | 1620 (HC = N), 1595 (C = N),<br>525 (M-N), 455 (M-O) | 28510 |

**Infrared spectra**

IR spectra of the Schiff bases showed the absence of bands at 1735 and 3420 $cm^{-1}$ due to carbonyl ν(C=O) and $\nu(NH_2)$ stretching vibrations and, instead, appearance of a strong new band at ~1635 $cm^{-1}$ assigned[30] to the azomethine ν(HC=N) linkage. The comparison of the infrared spectra of the Schiff bases with their metal chelates indicated that the Schiff bases were principally coordinated to the metal atom in three ways, thus representing the ligands as acting tridentately:

a) The band appearing at 1635 $cm^{-1}$ due to the azomethine, shifted to lower frequency by ~10-15 $cm^{-1}$ indicating[31] participation of the azomethine nitrogen in complexation.
b) The band at 1620 assigned to thiadiazole ring ν(C=N) nitrogen also shifted to lower frequency by ~10 $cm^{-1}$ which was indicative of the involvement of thiadiazole ring nitrogen in chelation.
c) A band appearing at 3425 $cm^{-1}$ assigned to ν(OH) in the Schiff base compounds was not found in the spectra of their metal complexes indicating deprotonation and coordination of the hydroxyl oxygen to the metal ion.
d) Further evidence of the coordination of these Schiff base compounds with the metal ions, was shown by the appearance of weak low frequency new bands at 525-530 and 455-460 $cm^{1}$ (Table 3). These were, assigned[32] to the metal-nitrogen ν(M-N) and metal-oxygen ν(M-O) respectively. These new bands were observable only in the spectra of the metal complexes and not in the spectra of its uncomplexed Schiff base compounds thus confirming participation of these hetero groups (O or N) in the coordination.

**NMR Spectra**

The NMR spectral data of Schiff bases as well as some of their Zn(II) complexes taken in DMSO-$d_6$ are listed in Tables 1 and 4. The Schiff bases exhibited signals due to all the expected protons in their expected region and have been identified from the integration curves found to be equivalent to the total number of protons deduced from their proposed structures. These were compared with the reported[33] signals of the known identical compounds and give further support for the compositions of these new Schiff bases as well as their complexes suggested by their IR and elemental analyses data. Comparison of the chemical shifts of the uncomplexed Schiff bases with those of the corresponding complexes show that some of the resonances are shifted upon complexation. In each case, the protons assigned due to heteroaromatic (HC=N), azomethine (HC=N), hydroxyl group (OH) and substituted aromatic were found at around δ 8.8, 7.4, 9.9 and 6.8-7.7 ppm in the spectra of the Schiff bases. The protons due to heteroaromatic, azomethine and substituted aromatic undergo shift towards downfield in the complexes indicating coordination of these groups with the metal atom. Also, protons due to hydroxyl group (OH) were found absent in the spectra of the complexes. The absence of these signals suggested[34] the deprotonation of the hydroxyl oxygen atom of the Schiff base on complexation. The same shifts were observed in the $^{13}C$ NMR spectral data of the Schiff bases and their complexes.

Table 4: NMR spectra of the Zn(II) complexes 10-12.

| Complex | $^{1}H$ NMR (DMSO-$d_6$) (ppm) | $^{13}C$ NMR (DMSO-$d_6$) (ppm) |
|---|---|---|
| 10 | 8.8 (s, 1H, heteroaromatic), 7.5 (s, 1H, CH=N), 6.8 (m, 1H, Ph), 7.2 (m, 1H, Ph), 7.4 (m, 1H, Ph), 7.7 (m, 1H, Ph). | 116.6, 118.3, 118.7 (aromatic), 131.2, 132.3, 160.7 (aromatic), 143.3, 156.4 (heteroaromatic), 165.3 (HC=N). |
| 11 | 8.9 (s, 1H, heteroaromatic), 7.7 (s, 1H, CH=N), 7.3 (m, 1H, Ph), 7.6 (m, 1H, Ph), 7.8 (m, 1H, Ph). | 116.7, 118.3, 118.8 (aromatic), 131.7, 132.5, 160.7 (aromatic), 143.4, 155.9 (heteroaromatic), 165.3 (HC=N). |
| 12 | 3.6 (s, 3H, $OCH_3$, 8.9 (s, 1H, heteroaro-matic), 7.8 (s, 1H, CH=N), 7.6 (m, 1H, Ph), 7.5 (m, 1H, Ph), 7.7 (m, 1H, Ph). | 31.8 ($OCH_3$), 116.7, 118.2, 118.7 (aromatic), 131.7, 132.3, 160.7 (aromatic), 143.4, 156.3 (heteroaromatic), 165.3 (HC=N). |

**Magnetic Moments and UV-Visible Spectra**

The room temperature magnetic moment of the solid cobalt(II) complexes was found lie in the range (4.2-4.7 B.M), indicative[35] of three unpaired electrons per Co(II) ion in an octahedral environment. The Cu(II) complexes showed $\mu_{eff}$ values in the range (1.4-1.6 B.M) indicative of one unpaired electron per Cu(II) ion suggesting[36] these complexes within the range consistent to spin-free distorted octahedral geometry. Similarly the Ni(II) complexes showed $\mu_{eff}$ values in the range (3.1-3.4 B.M), corresponding[36] to two unpaired electrons per Ni(II) ion for their ideal six-coordinated configuration. The Zn(II) complexes were all found diamagnetic.

The electronic spectra of the Co(II) chelates showed three bands observed at 8780-8815, 17560-18425 and 30210-30575 $cm^{-1}$ which may be assigned to $^4T_{1g} \rightarrow {}^4T_{2g}(F)$, $^4T_{1g} \rightarrow {}^3A_{2g}(F)$ and $^4T_{1g} \rightarrow {}^4T_{1g}(P)$ transitions respectively and are suggestive[37,38] of the octahedral geometry around the cobalt ions.

The Cu(II) complexes showed three absorption bands between 10 Dq band for a distorted octahedral geometry corresponding[39,40] to the transitions $^2E_g \rightarrow ^2T_{2g}$. The bands at 22152-22355 and 30550-30645 $cm^{-1}$ may be due to intra-ligand charge transfer transitions.

The Ni(II) complexes exhibited three spin-allowed bands at 9815-10145, 15945-16250 and 28540-29210 $cm^{-1}$ assignable[41,42] respectively, to the transitions $^3A_{2g}(F) \rightarrow ^3T_{2g}(F)(\nu_1)$, $^3A_{2g}(F) \rightarrow ^3T_{1g}(F)(\nu_2)$ and $^3A_{2g}(F) \rightarrow ^3T_{2g}(P)(\nu_3)$ which were characteristic of their octahedral geometry (Fig 2).

The diamagnetic zinc(II) complexes did not show any d-d bands and their spectra are dominated only by charge transfer bands. The charge transfer band at 28450-28510 $cm^{-1}$ was assigned[43] due to transition $^2E_g \rightarrow ^2T_{2g}$ possibly in an octahedral environment.

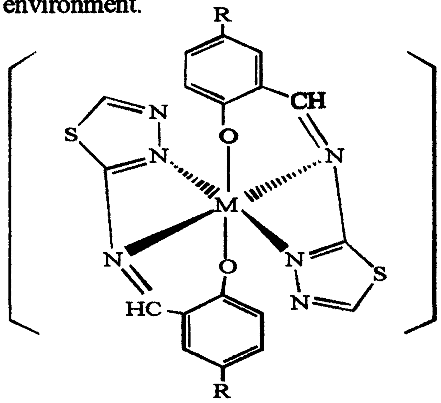

M=Co(II), Cu(II), Ni(II) or Zn(II)

(Fig. 2) Proposed Structure of the Metal(II) Complex

**Antibacterial Properties**

The title Schiff bases and their metal chelates were evaluated for their antibacterial activity against bacterial strains of *Escherichia coli (a), Staphylococcus aureus (b) and Pseudomonas aeruginosa (c)* (Table III). The compounds were tested at a concentration of 30 μg/0.01 mL in DMF solution using the paper disc diffusion method. The diameter of the susceptibility zones were measured in (mm). The results of which are shown in Table 4. The susceptibility zones measured were the clear zones around the discs killing the bacteria.

All the Schiff bases and their complexes individually exhibited varying degrees of inhibitory effects on the growth of the tested bacterial species. The antibacterial results evidently show that the activity of the Schiff base compounds became more pronounced when coordinated to the metal ions.

On the basis of these observations, it is claimed that chelation dominantly affects the biological behavior of the compounds that are potent against some bacterial strains. It is however, suspected that factors such as solubility, dipole moment and cell permeability mechanisms are certainly influenced by the presence of the metal ions.

Table III. Antibacterial Activity Data of the Schiff bases and its Metal(II) chelates

| Schiff base/ Chelate | Microbial Species a | b | c |
|---|---|---|---|
| $HL^1$ | ++ | + | ++ |
| $HL^2$ | ++ | ++ | ++ |
| $HL^3$ | +++ | ++ | +++ |
| 1 | +++ | +++ | ++ |
| 2 | +++ | ++ | +++ |
| 3 | +++ | +++ | +++ |
| 4 | ++++ | +++ | +++ |
| 5 | +++ | +++ | +++ |
| 6 | ++++ | ++ | +++ |
| 7 | ++ | +++ | + |
| 8 | +++ | +++ | +++ |
| 9 | +++ | +++ | +++ |
| 10 | +++ | +++ | ++ |
| 11 | ++ | +++ | ++++ |
| 12 | +++ | +++ | +++ |

a= *Escherichia coli*, b= *Staphylococcus aureus*, c= *Pseudomonas aeruginosa*
Inhibition zone diameter mm (% inhibition): +, 6-10 (27-45 %); ++, 10-14 (45-64 %); +++, 14-18 (64-82 %); ++++, 18-22 (82-100 %). Percent inhibition values are relative to inhibition zone (22 mm) of the most active compound with 100 % inhibition.

**ACKNOWLEDGEMENT**
The authors gratefully acknowledge the help of the Department of Pathology, Qaid-e-Azam Medical College, Bahawalpur, in undertaking the antibacterial studies.

**REFERENCES**

1. A. Y. Louie, and T. J. Meade, *Chem. Rev*, **99**, 2711 (1999).
2. a) A. Scozzafava, L. Menabuoni, F. Mincione, G. Mincione and C.T. Supuran, *Bioorg. Med. Chem. Lett*, **11**, 575 (2001); b) A. Scozzafava and C. T. Supuran *J. Med. Chem*, **43**, 3677 (2000).
3. a) R. J. P. Williams, *Quart. Rev*, **24**, 331 (1970); b) D. R. Williams, "*The Metals of Life*", Van Nostrand, London, (1971); c) R.J.P. William, "*Bioinorganic Chemistry*", American Chemical Society, Washington, (1971).
4. a) D. H. Brown, W. E. Smith, J. W. Teape and A. J. Lewis, *J. Med. Chem*, **23**, 729 (1980); b) R. D. Gillard, *Inorg. Chim. Acta. Rev*, **1**, 60 (1961); c) M. Dixon and E. C. Webb, "*Enzymes*", Green and Co, London, (1964); d) J. Schubert, *Sci. Amer*, **214**, 40 (1966).
5. C. Walsh, *Science*, **409**, 226 (2001).
6. I. Bertini, H. B. Gray, S. J, Lippard, J. S. Valentine, "*Bioinorganic Chemistry*", Univ. Science Books, Mill valey, CA, USA, (1994).
7. R. K. Watt and P. W. Ludden, *Cell. Mol. Life Sci* **56**, 604 (1999).
8. a) S. Kirschner, Y. K. Wei, D. Francis and D. Bergman, *J. Med. Chem*, **9**, 369 (1966), b) S. Kirschner, S. H. Kravitz and J. Mack, *J. Chem. Documentation*, **6**, 213 (1966).
9. a) J. A. Loo, T. P. Holler, J. Sanchez, R. Gogliotti, L. Maloney and M. D. Reily, *J. Med. Chem*, **39**, 4313 (1996); b) M. Huang, A. Maynard, J. A. Turpin, L. Graham, G. M. Janini, D. G. Covell and W. G. Rice, *J. Med. Chem*, **41**: 1371 (1998); c) W. Beerheide, M. M. Sim, Y. J. Tan, H. U. Bernard and A. E. Ting, *Bioorg. Med. Chem*, **8**: 2549 (2000).
10. S. E. Livingstone and J. D. Nolan and A. E. Mihkelson, *Inorg. Chem*, **9**, 2545 (1970).
11. S. E. Livingstone and J. D. Nolan, *Inorg. Chem*, **7**, 1447 (1968).
12. M. E. Heim, "*Metal Complexes in Chemotherapy*", Verlag Chemie, Weinheim, (1993).
13. J. R. J. Sorenson, *J. Appli. Nutr*, **32**, 4 (1980).
14. J. R. J. Sorenson, "*Inflammatory Diseases and Copper*", Human Press, NJ, (1982).
15. R. Nagar and G. Mohan, *J. Inorg. Biochem*, **42**, 9 (1991).
16. S. Oga, S. F. Taniguchi, R. Najjar and A. R. Souza, *J. Inorg. Biochem*, **41**, 45 (1991).
17. M. Barboiu, M. Cimpoesu, C. Guran and C. T. Supuran, *Metal-Based Drugs*, **3**, 227 (1996).
18. N. S. Habib and S. A. E. Shams, *J. Pharm. Sci*, **45**, 310 (1977).
19. J. Hiendl and E. K. Schroeder, *Eur. J. Med. Chem.*, **10**, 121 (1975).
20. a) A. S. Yanni, *J. Indian. Chem*, **68**, 529 (1991); b) K. Takatori, T. Hasagawa and K. J. Nakano, *Microbial. Immunol*, **29**, 1237 (1985).
21. a) A. Mastrolorenzo and C.T. Supuran, *Metal-Based Drugs*, **7**, 49 (2000); b) A. Mastrolorenzo, A. Scozzafava and C.T. Supuran, *J. Enz. Inhib*, **15**, 517 (2000); c) A. Mastrolorenzo, A. Scozzafava and C.T. Supuran, *Eur. J. Pharm. Sci.*, **11**, 99 (2000).
22. K. Miyamato, R. Koshiura, H. Yokoi, C. Mori, T. Hasegawa and K. Takotri, *Chem. Pharm. Bull*, **33**, 5126 (1985).
23. a) I. S. Grasso, A. Chimirri, P. Monforte and G. Fenech, *Pharmaco*, **39**, 505 (1984), b) N. H. Jayaram, H. Zhen, E. N. Weining and M. Kamran, *Cancer. Res*, **53**, 2344 (1995).
24. C.T. Supuran, A. Scozzafava *Eur. J. Med. Chem*, **35**, 867(2000).
25. K. Y. R. Zee-Cheng and C. C-Cheng, *J. Med. Chem*, **28**, 1216 (1985).
26. a) C. T. Supuran, A. Scozzafava and A. Mastrolorenzo, *Exp. Opin. Ther. Patents* **11**, 221 (2001); b) Z. H. Chohan and S. K. A. Sherazi, *Synth. React. Inorg. Met.-Org. Chem*, **29**, 105 (1999).
27. Z. H. Chohan and S. Kausar, *Metal-Based Drugs*, **7**, 17 (2000).
28. Z. H. Chohan and M. Praveen, *Appl. Organomet. Chem*, **14**, 376 (2000).
29. W. J. Geary, *Coord. Chem. Rev*, **7**, 81 (1971).
30. L. J. Bellamy, "*The Infrared Spectra of Complex Molecules*", 3rd Ed, Methuen, London, (1966).
31. M. Yongxiang, Z. Zhengzhi, M. Yun and Z. Gang, *Inorg. Chim. Acta*, **165**, 185 (1989).
32. K. Nakamoto, "*Infrared Spectra of Inorganic and Coordination Compounds*", 2nd Ed, Wiley Interscience, New York, (1970).

33. D. H. Williams and I. Fleming, "*Spectroscopic Methods in Organic Chemistry*", 4th Ed, Mc Graw Hill, London, (1989).
34. Z. Hong-Yun, C. Dong-Li, C. Pei-Kun, C. De-Ji, C. Guang-Xia and Z. Hong-Quan, *Polyhedron*, **11**, 2313 (1992).
35. B. N. Figgis, "*Introduction to Ligand Fields*", J. Wiley, New York, (1976).
36. B. P. Lever, "*Inorganic Electronic Spectroscopy*", Elsevier, Amsterdam, (1984).
37. D. Liehr, *J. Phys. Chem*, **67**, 1314 (1967).
38. T. M. Dunn, J. Lewis and R. C. Wilkins, "*The Visible and Ultraviolet Spectra of Complex Compounds in Modern Coordination Chemistry*", Interscience, New York, (1960).
39. A. B. P. Lever, J. Lewis and R. S. Nyholm, *J. Chem. Soc*, 4761 (1964).
40. R. L. Carlin,., "*Transition Metal Chemistry*", Ed, R. L. Carlin, Vol 1, Marcel Decker, New York, (1965).
41. D. W. Meek, R. S. Drago and T. S. Piper, *Inorg. Chem*, **1**, 285 (1962).
42. R. S..Drago, "*Physical Methods in Inorganic Chemistry*", Reinhold, New York, (1965).
43. B. N. Figgis and J. Lewis, *Prog. Inorg. Chem*, **6**, 87 (1964).

**Accepted: February 14, 2001 – Accepted: March 12, 2001 –**
**Accepted in publishable format: May 8, 2001**

